# *Lactobacillus apinorum* sp. nov., *Lactobacillus mellifer* sp. nov., *Lactobacillus mellis* sp. nov., *Lactobacillus melliventris* sp. nov., *Lactobacillus kimbladii* sp. nov., *Lactobacillus helsingborgensis* sp. nov. and *Lactobacillus kullabergensis* sp. nov., isolated from the honey stomach of the honeybee *Apis mellifera*

**DOI:** 10.1099/ijs.0.059600-0

**Published:** 2014-09

**Authors:** Tobias C. Olofsson, Magnus Alsterfjord, Bo Nilson, Èile Butler, Alejandra Vásquez

**Affiliations:** Department of Laboratory Medicine, Medical Microbiology, Lund University, Sölvegatan 23, SE-223 62 Lund, Sweden

## Abstract

We previously discovered a symbiotic lactic acid bacterial (LAB) microbiota in the honey stomach of the honeybee *Apis mellifera*. The microbiota was composed of several phylotypes of *Bifidobacterium* and *Lactobacillus*. 16S rRNA gene sequence analyses and phenotypic and genetic characteristics revealed that the phylotypes isolated represent seven novel species. One grouped with *Lactobacillus kunkeei* and the others belong to the *Lactobacillus buchneri* and *Lactobacillus delbrueckii*subgroups of *Lactobacillus*. We propose the names *Lactobacillus apinorum* sp. nov., *Lactobacillus mellifer* sp. nov., *Lactobacillus mellis* sp. nov., *Lactobacillus melliventris* sp. nov., *Lactobacillus kimbladii* sp. nov., *Lactobacillus helsingborgensis* sp. nov. and *Lactobacillus kullabergensis* sp. nov. for these novel species, with the respective type strains being Fhon13N^T^ ( = DSM 26257^T^ = CCUG 63287^T^), Bin4N^T^ ( = DSM 26254^T^ = CCUG 63291^T^), Hon2N^T^ ( = DSM 26255^T^ = CCUG 63289^T^), Hma8N^T^ ( = DSM 26256^T^ = CCUG 63629^T^), Hma2N^T^ ( = DSM 26263^T^ = CCUG 63633^T^), Bma5N^T^ ( = DSM 26265^T^ = CCUG 63301^T^) and Biut2N^T^ ( = DSM 26262^T^ = CCUG 63631^T^).

In 2005, we discovered a symbiotic lactic acid bacterial (LAB) microbiota in the honey stomach of the Western honeybee, *Apis mellifera* (Olofsson & Vásquez, 2008). The honey stomach is a central organ in the honeybee’s food production, used for the collection of nectar and its transport to the hive. This previously unknown microbiota is composed of several phylotypes within the genera *Lactobacillus* and *Bifidobacterium* and plays a key role in the production of honey (Olofsson & Vásquez, 2008; Vásquez *et al.*, 2012) and bee bread (Vásquez & Olofsson, 2009), food that is stored long-term and consumed by both adult honeybees and larvae. Our recent studies have also shown that the LAB microbiota is consistent across the native and introduced *A. mellifera* range (Olofsson & Vásquez, 2008; Olofsson *et al.*, 2011; Vásquez *et al.*, 2009) and present similarly in all recognized honeybee species (Apini) as well as in stingless bee species (Meliponini) (Vásquez *et al.*, 2012). After our discovery, many research colleagues around the world have also found different or identical phylotypes of the genus *Lactobacillus* originating in the honey stomach of honeybees and their food (see Table 3). These phylotypes could be regarded as possible subspecies in the future. Besides its importance in the honeybee’s food production and preservation, this highly co-evolved microbiota has shown a protective action against severe bee pathogens (Forsgren *et al.*, 2010; Vásquez *et al.*, 2012) and bacteria present in nectar by producing active proteins (Butler *et al.*, 2013).

In the present study, the Western honeybee subspecies *A. mellifera mellifera* was sampled for LAB. Carl von Linné named this subspecies in 1758, at a time when it was free living in Europe. Today, this subspecies is protected, since it is threatened by extinction. The collected bees originated from the same apiary in a protected area in Hammerdal, Jämtland, in northern Sweden, and they are part of a conservation project in Sweden called Nordbi. The 16S rRNA genes were sequenced from a total of 168 isolates sampled from different honeybee crops and honeybee foods. Ninety-one of these isolates were related most closely to the genus *Lactobacillus* but distantly related to any existing species of the genus *Lactobacillus*. Fourteen unique isolates were selected for further analyses to determine their novelty as members of novel species of the genus *Lactobacillus*: strains Fhon13N^T^ ( = DSM 26257^T^ = CCUG 63287^T^), Bin4N^T^ ( = DSM 26254^T^ = CCUG 63291^T^), Hon2N^T^ ( = DSM 26255^T^ = CCUG 63289^T^), Hma8N^T^ ( = DSM 26256^T^ = CCUG 63629^T^), Hma2N^T^ ( = DSM 26263^T^ = CCUG 63633^T^), Bma5N^T^ ( = DSM 26265^T^ = CCUG 63301^T^), Biut2N^T^ ( = DSM 26262^T^ = CCUG 63631^T^), Kvahm3N ( = DSM 26315 = CCUG 63288), Bin4 ( = DSM 26316 = CCUG 65819), H1hs38N ( = DSM 26313 = CCUG 63290), Hma8 ( = DSM 26312 = CCUG 65820), H6hs28N ( = DSM 26318 = CCUG 63634), H4bb18N ( = DSM 26317 = CCUG 63635) and H6hs21N ( = DSM 26314 = CCUG 63632). These strains have been deposited with the Deutsche Sammlung von Mikroorganismen und Zellkulturen GmbH (DSMZ) and the Culture Collection, University of Gothenburg (CCUG).

Honey stomachs of incoming foragers, fresh honey, corbicular bee pollen and bee bread were collected as described previously (Olofsson & Vásquez, 2008; Vásquez & Olofsson, 2009). Unless otherwise stated, pure bacterial isolates were obtained under anaerobic cultivation (AnaeroGen Compact System; Oxoid) at 35 °C on De Man, Rogosa and Sharpe (MRS) agar plates (Oxoid) or in MRS broth supplemented with 0.1 % l-cysteine (Sigma) and 2.0 % fructose (Merck). The isolates were incubated for 3 days. PCR amplification, 16S rRNA gene sequencing, identification and phylogenetic analysis (Fig. 1) were performed according to Olofsson & Vásquez (2008). In addition, the 16S rRNA gene sequences (1450 bp) were also checked against the Ribosomal Database Project II (RDP) software (http://rdp.cme.msu.edu/).

**Fig. 1.**
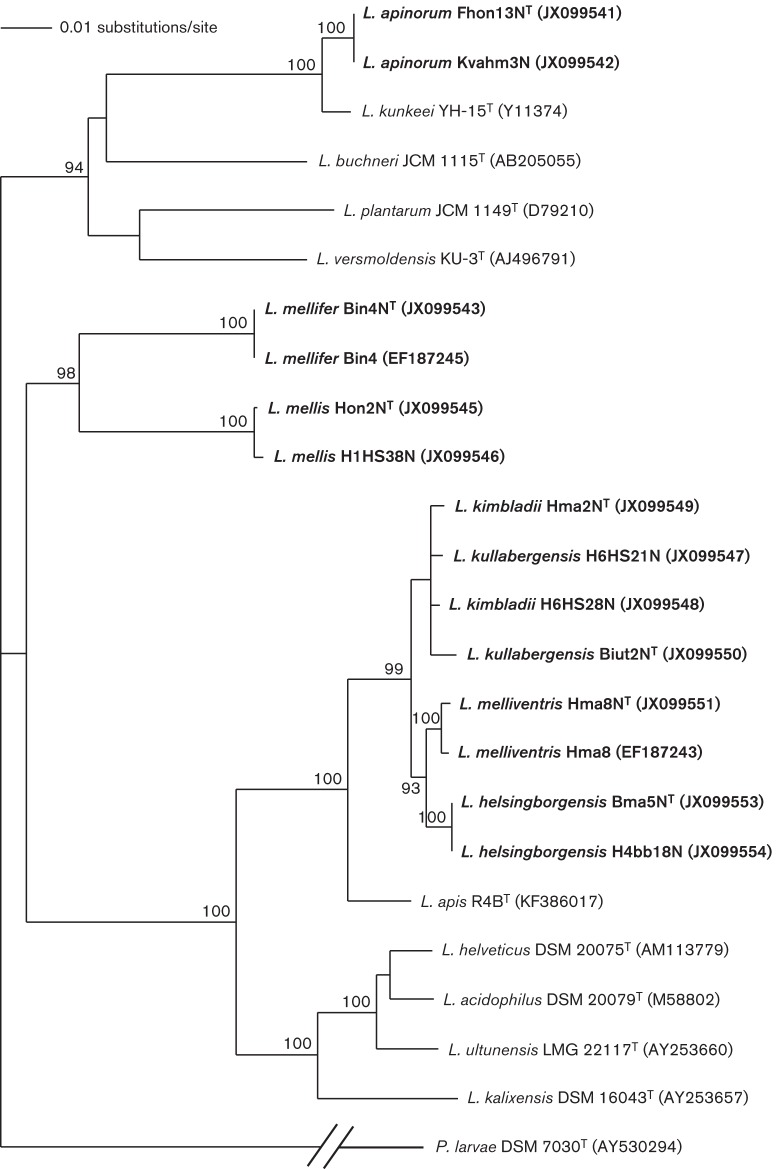
Phylogenetic tree derived from 16S rRNA gene sequence analyses, showing the relationship of the novel species to members of the *L. buchneri* and *L. delbrueckii*subgroups of the lactobacilli. The sequence of *Paenibacillus larvae*DSM 7030^T^ served as an outgroup. Approximately 1450 nt from each sequence were used for the alignment. Bar, 0.01 substitutions per nucleotide position. Numbers indicate bootstrap percentages for branch points. GenBank accession numbers are given in parentheses.

The genome sequences of the proposed type strains Fhon13N^T^, Bin4N^T^, Hon2N^T^, Hma2N^T^, Bma5N^T^, Hma8N^T^ and Biut2N^T^ (Butler *et al.*, 2013) and *Lactobacillus kunkeei* YH-15^T^ were used for various analyses in the present study.

Cell morphologies and spore-forming abilities of each isolate were examined by phase-contrast microscopy and by SEM as described previously (Vásquez *et al.*, 2012). Determination of Gram staining reactions was performed using a Gram staining kit (bioMérieux). Sugar fermentation patterns and aesculin hydrolysis were assessed using the API 50 CHL system (bioMérieux) in duplicate after 5 days of incubation at 35 °C.

Enzyme activities of the LAB strains were measured using the API ZYM strip (bioMérieux) as described by the manufacturer. Each LAB strain was grown on MRS agar (supplemented with 2 % fructose and 0.1 % l-cysteine) at 35 °C for 72 h prior to testing.

Analyses of cellular fatty acids and polar lipids were carried out by the DSMZ according to previously described methods (Bligh & Dyer, 1959; Kämpfer & Kroppenstedt, 1996; Miller, 1982; Tindall *et al.*, 2007). After cultivation, bacterial cells were collected by centrifugation and lyophilized.

Lactic acid configuration was determined using a test kit from Boehringer Mannheim. Catalase activity was determined by transferring fresh colonies from MRS agar to a glass slide and adding 5 % H_2_O_2_ (bioMérieux). Whole genomes were searched for genes coding for catalase using the ERGO database (Integrated Genomics) (Overbeek *et al.*, 2003) and blastall 2.2.26 (on the NCBI website) (Altschul *et al.*, 1990). Homofermentative and heterofermentative characterization was done by growing the bacteria in MRS broth lacking citrate (Schillinger & Lücke, 1987), with inverted Durham tubes to observe CO_2_ production from glucose. Growth at various pH and temperatures was determined adjusting MRS broth with HCl and NaOH and cultivation of the bacteria on MRS plates at various temperatures. Aerobic growth was determined by cultivating the bacteria on MRS plates.

For MALDI-TOF MS protein profiling, bacterial cell extracts were prepared from fresh colonies using the ethanol/formic acid extraction procedure as recommended by the manufacturer (Bruker Daltonik) and described previously (Mellmann *et al.*, 2008). Extracts were pipetted onto a clean, polished steel target plate and, once air-dried, overlaid with a saturated α-cyano-4-hydroxycinnamic acid (CHCA) matrix solution. Automatic mass spectrum collections were acquired in linear positive mode using an Ultaflextreme MALDI-TOF/TOF mass spectrometer and the software flexControl 3.3 and MALDI Biotyper RTC 3.0 (Bruker Daltonics) with standard settings. The MALDI Biotyper 3.0 offline client software was used for mass spectrum-based identification and classification of the strains against the Bruker Daltonics database containing main spectra (MSP). An internal MSP library consisting of the described bacterial strains was created according to Bruker’s standard procedures using MALDI Biotyper. This library can be used in combination with the Bruker database or as a stand-alone library. The MSP dendrogram was calculated using the MALDI Biotyper and the settings used were correlation as distance measure, and the average linkage algorithm. The distances were normalized to between 0 and 1000.

To determine the peptidoglycan structure of the bacterial cell walls, we checked for the ability to produce *meso*-diaminopimelic acid (m-DAP), searching the whole-genome sequences using the ERGO database (Integrated Genomics) (Overbeek *et al.*, 2003) and blastall 2.2.26 (NCBI) (Altschul *et al.*, 1990). The presence of the m-DAP synthesis pathway does not prove that the bacterial cell wall contains m-DAP as its diamino acid; for instance, it can be used as a pathway to produce lysine. To determine the cell-wall composition further, genes coding for enzymes specific for building up the peptides in the peptidoglycan were identified in whole-genome sequences. Genome analyses, together with traditional peptidoglycan analyses (Schumann, 2011), were performed to determine the peptidoglycan structure. DNA–DNA relatedness values were determined at the DSMZ. Using the procedure of Cashion *et al.* (1977), the DNA was isolated by chromatography on hydroxyapatite. DNA–DNA hybridizations were carried out as described by De Ley *et al.* (1970), with the modifications as described by Huss *et al.* (1983), using a Varian Cary 100 Bio UV/Vis spectrophotometer equipped with a Peltier-controlled 6×6 multicell changer and a temperature controller with *in situ* temperature probe (BioTech). Mean values were calculated from duplicates.

As a complement to DNA–DNA hybridization, the average nucleotide identity (ANI) of the total genomic sequence shared between two strains (pairwise comparison) was determined according to Goris *et al.* (2007). Whole-genome sequences in a pairwise comparison were cut into consecutive 1020 bp fragments. One of the cut genome sequences was used as the query sequence, while the other sequence was used as the subject. The 1020 bp fragments were aligned using the blast 2.2.26 (ANIb) algorithm (Altschul *et al.*, 1990). Whole query and subject genomes were aligned with nucmer in MUMmer 3.23 software (ANIm) (Kurtz *et al.*, 2004). ANI values were calculated using JSpecies 1.2.1 software (Goris *et al.*, 2007). The DNA G+C content was determined based on whole-genome sequence analysis.

The major fatty acids detected in strains Fhon13N^T^, Bin4N^T^, Hon2N^T^, Hma8N^T^, Hma2N^T^, Bma5N^T^ and Biut2N^T^ were C_18 : 1_ω9*c* (8.55, 44.57, 42.81, 51.62, 40.48, 53.11 and 47.22 %, respectively), C_16 : 0_ (44.20, 16.29, 17.34, 16.39, 26.73, 15.71 and 20.78 %), summed unknown 18.846/C_19 : 1_ω6*c*/C_19 : 0_ cyclo ω10*c/*C_19_ω6 (9.64, 14.74, 18.04, 10.56, 14.73, 9.28 and 12.05 %) and C_19 : 0_ cyclo ω8*c* (25.36, 11.32, 7.20, 7.20, 6.41, 7.83 and 6.21 %).

Phylogenetic analysis (Fig. 1) of the 16S rRNA gene sequences placed strain Fhon13N^T^ in a group with *L. kunkeei* (Felis & Dellaglio, 2007). The 16S rRNA gene sequence of Fhon13N^T^ was most closely related to that of the type strain of *L. kunkeei*, with 98.9 % similarity. Strains Bin4N^T^ and Hon2N^T^ were placed in the *Lactobacillus buchneri*subgroup of the lactobacilli (Felis & Dellaglio, 2007). The 16S rRNA gene sequences of strains Bin4N^T^ and Hon2N^T^ were most closely related to those of the type strains of *L. buchneri*, *L. versmoldensis* and *L. plantarum*, with ≤88.9 and ≤89.4 % similarity, respectively. Strains Hma8N^T^, Hma2N^T^, Bma5N^T^ and Biut2N^T^ were placed in the *Lactobacillus delbrueckii* subgroup of the lactobacilli (Felis & Dellaglio, 2007). The 16S rRNA gene sequences of strains Hma8N^T^, Hma2N^T^, Bma5N^T^ and Biut2N^T^ were most closely related to those of the type strains of *Lactobacillus acidophilus*, *L. helveticus* and *L. ultunensis*, with ≤92.4, ≤92.7, ≤90.0 and ≤86.1 % similarity, respectively.

Because of the high 16S rRNA gene sequence similarity between Fhon13N^T^ and the type strain of *L. kunkeei*, ANI analysis and DNA–DNA hybridization were performed. The levels of ANIb and DNA–DNA relatedness between Fhon13N^T^ and *L. kunkeei* CCUG 53901^T^ (Table 2) were well below the threshold of the recommended ANI of 95 % (Goris *et al.*, 2007) and the recommendations of a threshold value of 70 % DNA–DNA relatedness for the definition of bacterial species (Wayne *et al.*, 1987).

Strains Hma8N^T^, Hma2N^T^, Biut2N^T^ and Bma5N^T^ also showed high 16S rRNA gene sequence similarity. Strain Hma8N^T^ showed 98.3, 98.5 and 99.0 % similarity to strains Hma2N^T^, Biut2N^T^ and Bma5N^T^, respectively, Hma2N^T^ showed 98.3, 99.1 and 98.5 % similarity, respectively, to strains Hma8N^T^, Biut2N^T^ and Bma5N^T^, strain Bma5N^T^ showed 99.0, 98.2 and 98.5 % similarity, respectively, to Hma8N^T^, Biut2N^T^ and Hma2N^T^, and, finally, strain Biut2N^T^ showed 98.5, 98.2 and 99.1 % similarity, respectively, to strains Hma8N^T^, Bma5N^T^ and Hma2N^T^. Therefore, ANI analysis and DNA–DNA hybridization were performed between the four strains. The levels of ANIb and DNA–DNA relatedness between strains Hma8N^T^, Hma2N^T^, Biut2N^T^ and Bma5N^T^ (Table 2) were well below the threshold of the recommended ANI of 95 % (Goris *et al.*, 2007) and the recommendations of a threshold value of 70 % DNA–DNA relatedness for the definition of bacterial species (Wayne *et al.*, 1987).

The 16S rRNA gene sequence of the reference strain Kvahm3N showed 100 % similarity to that of Fhon13N^T^. In the MSP dendrogram, Fhon13N^T^ grouped closely together with the reference strain Kvahm3N, and both were well separated from the closest type strain, *L. kunkeei* CCUG 53901^T^ (Fig. 2). According to this analysis, strains Fhon13N^T^ and Kvahm3N represent a novel species of the genus *Lactobacillus*, for which we propose the name *Lactobacillus apinorum* sp. nov.

**Fig. 2.**
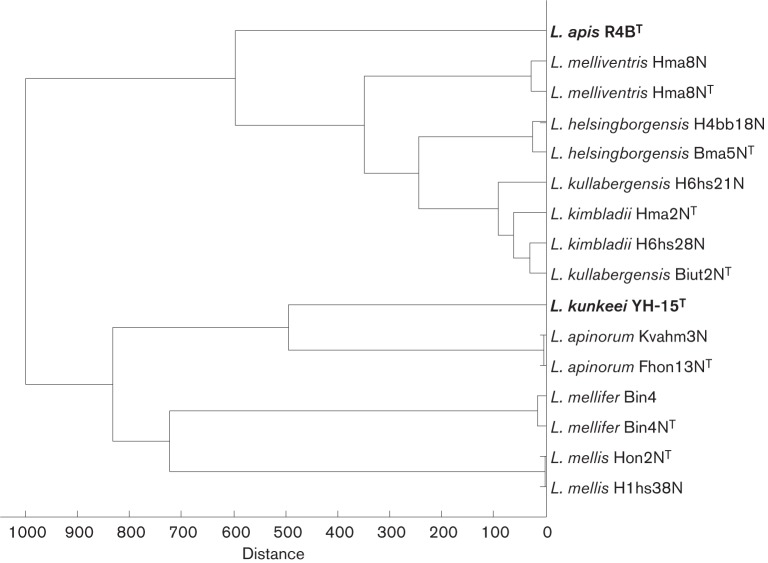
Main spectrum (MSP) dendrogram derived from MALDI-TOF MS protein profiling of strains of *L. apinorum* sp. nov., *L. mellifer* sp. nov., *L. mellis* sp. nov., *L. melliventris* sp. nov., *L. kimbladii* sp. nov., *L. helsingborgensis* sp. nov., *L. kullabergensis* sp. nov. and *L. apis* and *L. kunkeei* as the most closely related reference strains (in bold).

The 16S rRNA gene sequence of the reference strain Bin4 showed 99.9 % similarity to that of Bin4N^T^. In the MSP dendrogram, Bin4N^T^ grouped closely together with the reference strain Bin4, and both were well separated from the other proposed type strains (Fig. 2). According to this analysis, strains Bin4N^T^ and Bin4 represent a second novel species of the genus *Lactobacillus*, for which we propose the name *Lactobacillus mellifer* sp. nov.

The 16S rRNA gene sequence of the reference strain H1hs38N showed 99.8 % similarity to that of Hon2N^T^. In the MSP dendrogram, Hon2N^T^ grouped close together with the reference strain H1hs38N, and both were well separated from the other proposed type strains (Fig. 2). According to this analysis, strains Hon2N^T^ and H1hs38N represent another novel species of the genus *Lactobacillus*, for which we propose the name *Lactobacillus mellis* sp. nov.

The 16S rRNA gene sequence of the reference strain H6hs25N showed 100 % similarity to that of Hma8N^T^. In the MSP dendrogram, Hma8N^T^ grouped close together with the reference strain Hma8, and both were well separated from the closest type strain, *Lactobacillus apis* R4B^T^, and the other proposed type strains (Fig. 2). According to this analysis, strains Hma8N^T^ and H6hs25N represent a fourth novel species of the genus *Lactobacillus*, for which we propose the name *Lactobacillus melliventris* sp. nov.

The 16S rRNA gene sequence of the reference strain H6hs28N showed 99.6 % similarity to that of Hma2N^T^. In both the phylogenetic tree (Fig. 1) and the MSP dendrogram (Fig. 2), Hma2N^T^ grouped close together with the reference strain H6hs28N, but the reference strain also grouped close to the proposed novel type strain Biut2N^T^ and the reference strain H6hs21N; however, these results varied with settings in the respective software, especially in the phylogenetic tree. Results from API 50 tests (Table 1) clearly show the relatedness of the reference strains to their respective proposed type strains. Both strains were well separated from the closest type strain, *L. apis* R4B^T^, and the other proposed type strains. According to this analysis, strains Hma2N^T^ and H6hs28N represent another novel species of the genus *Lactobacillus*, for which we propose the name *Lactobacillus kimbladii* sp. nov.

**Table 1.** Characteristics that differentiate the proposed novel species of *Lactobacillus* from some close phylogenetic relatives Strains: 1, *L. kunkeei* CCUG 53901^T^; 2, *L. apinorum* sp. nov. Fhon13N^T^; 3, *L. apinorum* sp. nov. Kvahm3N; 4, *L. mellis* sp. nov. Hon2N^T^; 5, *L. mellis* sp. nov. H1hs38N; 6, *L. mellifer* sp. nov. Bin4N^T^; 7, *L. mellifer* sp. nov. Bin4; 8, *L. kalixensis* (Roos *et al.*, 2005); 9, *L. ultunensis* (Roos *et al.*, 2005); 10, *L. apis* (Killer *et al.*, 2014); 11, *L. melliventris* sp. nov. Hma8N^T^; 12, *L. melliventris* sp. nov. Hma8; 13, *L. helsingborgensis* sp. nov. Bma5N^T^; 14, *L. helsingborgensis* sp. nov. H4bb18N; 15, *L. kimbladii* sp. nov. Hma2N^T^; 16, *L. kimbladii* sp. nov. H6hs28N; 17, *L. kullabergensis* sp. nov. Biut2N^T^; 18, *L. kullabergensis* sp. nov. H6hs21N. Biochemical tests were performed using API CH50 and API ZYM systems (bioMérieux). +, Positive; w, weakly positive; −, negative; nd, not determined. All strains are positive for production of acid from d-glucose.

Characteristic	1	2	3	4	5	6	7	8	9	10	11	12	13	14	15	16	17	18
**Production of acid from:**																		
l-Arabinose	−	−	−	−	w	−	w	−	−	−	−	−	−	−	−	−	−	−
Ribose	−	−	−	−	−	−	+	−	−	−	−	−	−	−	−	−	−	−
d-Xylose	−	−	−	−	−	−	w	−	−	−	−	−	−	−	−	−	−	−
d-Galactose	+	−	−	−	−	−	−	+	+	−	−	−	w	−	−	+	−	−
d-Fructose	+	+	+	+	+	+	+	+	+	+	w	+	w	+	+	+	+	+
d-Mannose	−	−	−	−	−	−	+	+	+	+	+	+	+	+	+	+	−	−
l-Sorbose	−	−	−	−	−	−	−	−	−	−	−	−	+	−	−	−	−	−
Dulcitol	−	−	−	−	−	−	−	−	−	−	−	−	w	−	−	−	−	−
d-Mannitol	+	−	−	−	+	−	−	+	−	−	−	−	−	−	−	−	−	−
d-Sorbitol	−	−	−	−	−	−	−	−	−	−	−	−	+	+	−	−	−	−
Methyl α-d-glucoside	−	−	−	−	−	−	−	−	−	−	−	−	w	−	−	−	−	−
*N*-Acetylglucosamine	−	−	−	−	−	−	−	+	+	−	−	−	+	+	+	+	−	−
Amygdalin	−	−	−	−	−	−	−	+	−	w	−	−	w	+	−	−	−	w
Arbutin	−	−	−	+	−	+	+	+	+	+	−	+	w	−	+	+	−	−
Aesculin	−	−	−	+	+	+	+	+	+	+	+	+	+	+	+	+	−	+
Salicin	−	−	−	+	w	w	w	+	+	+	−	−	+	+	+	+	+	+
Cellobiose	−	−	−	−	−	−	−	+	+	−	−	−	−	−	−	−	−	−
Maltose	−	−	−	−	−	−	−	+	+	−	−	−	−	−	w	−	−	−
Lactose	−	−	−	−	−	−	w	+	+	−	−	−	−	−	−	−	−	−
Melibiose	−	−	−	−	−	−	+	+	w	−	−	−	−	w	−	−	−	−
Sucrose	+	−	−	−	−	−	w	+	+	−	−	−	+	−	−	−	−	−
Trehalose	−	−	−	−	−	−	+	+	+	w	−	−	−	w	−	−	+	−
Raffinose	+	−	−	−	−	−	−	+	+	−	−	−	w	−	−	−	−	−
Starch	−	−	−	−	−	−	−	+	+	−	−	−	−	−	−	−	−	−
Gentiobiose	−	−	−	−	−	−	−	+	+	−	+	−	−	−	−	w	+	−
d-Tagatose	−	−	−	−	−	−	+	−	−	−	w	−	−	−	+	−	−	−
d-Arabitol	−	−	−	−	−	+	w	−	−	−	−	−	−	−	−	−	−	−
**Enzyme activities**																		
Alkaline phosphatase	+	−	nd		nd		nd	nd	nd	−	−	nd	−	nd	−	nd	−	nd
Esterase (C4)	−	−	nd	+	nd	+	nd	nd	nd	w	+	nd	+	nd	−	nd	w	nd
Esterase lipase (C8)	−	−	nd	+	nd	+	nd	nd	nd	−	−	nd	−	nd	−	nd	−	nd
Leucine arylamidase	+	+	nd	+	nd	+	nd	nd	nd	+	+	nd	+	nd	+	nd	+	nd
Valine arylamidase	+	+	nd	+	nd	+	nd	nd	nd	+	+	nd	+	nd	+	nd	+	nd
Cystine arylamidase	−	w	nd	+	nd	+	nd	nd	nd	−	+	nd	−	nd	w	nd	−	nd
Acid phosphatase	+	+	nd	+	nd	+	nd	nd	nd	+	+	nd	+	nd	+	nd	+	nd
Naphthol-AS-BI-phosphohydrolase	+	+	nd	+	nd	+	nd	nd	nd	+	+	nd	+	nd	+	nd	+	nd
β-Galactosidase	−	−	nd	−	nd	−	nd	nd	nd	w	−	nd	+	nd	−	nd	−	nd
α-Glucosidase	−	−	nd	−	nd	−	nd	nd	nd	−	+	nd	−	nd	−	nd	−	nd
β-Glucosidase	−	−	nd	+	nd	+	nd	nd	nd	+	+	nd	+	nd	−	nd	+	nd
*N*-acetyl-β-glucosaminidase	−	−	nd	−	nd	−	nd	nd	nd	+	w	nd	−	nd	−	nd	w	nd

**Table 2.** Results from DNA–DNA hybridization and ANIb showing individual genome relatedness between closely related strains DNA–DNA hybridization results are given as percentages above the diagonal. ANIb values are given below the diagonal. nd, Not determined.

Strain	1	2	3	4	5	6
1. *L. kimbladii* Hma2N^T^	−	40.3	nd	53.1	44.5	nd
2. *L. helsingborgensis* Bma5N^T^	82.3	−	nd	50.4	11.8	nd
3. *L. kullabergensis* Biut2N^T^	nd	nd	−	nd	33.9	nd
4. *L. apinorum* Fhon13N^T^	92.4	82.5	nd	−	nd	37.4
5. *L. melliventris* Hma8N^T^	85.0	83.5	84.4	nd	−	nd
6. *L. kunkeei* CCUG 53901^T^	nd	nd	nd	79.1	nd	−

**Table 3.** Sequences of strains or clones of the genus *Lactobacillus* available in GenBank from different studies of the honeybee microbiota For each proposed type strain, representative closely related sequences are displayed. GenBank accession numbers are given in parentheses.

Sequence	Source	Sequence length (bp)	Similarity (%)
***L. apinorum* Fhon13N^T^ (JX099541)**			
kvahm3N (JX099542)	*A. mellifera mellifera* (honey stomach)	1445	100
fhon13 (HM534758)	*A. mellifera* Buckfast (fresh honey)	1446	100
bbt11 (HM534856)	*A. mellifera scutellata* (bee bread)	953	100
H8-7-4MCO2 (KF599250)	*A. mellifera* (honey stomach)	1403	100
hmto18 (HM534755)	*A. koshevnikovi* (honey stomach)	1446	100
hmto2 (HM534754)	*A. cerana* (honey stomach)	1446	100
hmto23 (HM534756)	*A. nuluensis* (honey stomach)	1446	100
honto4 (HM534757)	*A. laboriosa* (honey stomach)	1446	100
***L. mellis* Hon2N^T^ (JX099545)**			
H1hs38N (JX099546)	*A. mellifera mellifera* (honey stomach)	1435	99.9
Hon2 (EF187244)	*A. mellifera* Buckfast (fresh honey)	1407	99.7
bbr2 (HM534851)	*A. mellifera scutellata* (bee bread)	953	99.9
SHOG706 (HM113315)	*A. mellifera* (gut)	1395	99.7
H8-5-4MCO2 (KF599243)	*A. mellifera* (honey stomach)	1373	99.9
AcjLac3 (AB810024)	*A. cerana japonica* (digestive tract)	1487	99.7
Afpoto14 (HM534811)	*A. florea* (bee pollen)	1435	99.8
Aahmro15 (HM534812)	*A. andreniformis* (honey stomach)	1435	99.8
Mboho2r2 (HM534813)	*Meliponula bocandei* (fresh honey)	1435	99.8
***L. mellifer* Bin4N^T^ (JX099543)**			
Bin4 (EF187245)	*A. mellifera* Buckfast (honeybee)	1407	100
hmt5 (HM534848)	*A. mellifera monticola* (honey stomach)	750	99.0
Adhmto19 (HM534810)	*A. dorsata* (honey stomach)	1438	99.7
Adhmto1 (HM534809)	*A. dorsata* (honey stomach)	1438	99.8
73-15 (HE613304)	*A. mellifera carnica* (hindgut)	1371	99.4
SHOA452 (HM111914)	*A. mellifera* (abdomen)	1370	98.0
***L. helsingborgensis* Bma5N^T^ (JX099553)**			
H4bb18N (JX099554)	*A. mellifera mellifera* (bee bread)	1438	100
Bma5 (EF187242)	*A. mellifera* Buckfast (honey stomach)	1410	99.9
Bbr17 (HM534854)	*A. mellifera scutellata* (bee bread)	953	99.9
1F1 (AY667701)	*A. mellifera* (faeces)	1479	99.3
2L1 (AY667699)	*A. mellifera* (gut)	1456	99.5
SHOG703 (HM113313)	*A. mellifera* (gut)	1398	99.8
HBG-B1V1-3 (DQ837636)	*A. mellifera* (gut)	1372	99.9
Afpoto7 (HM534804)	*A. florea* (bee pollen)	1438	99.9
***L. melliventris* Hma8N^T^ (JX099551)**			
Hma8 (EF187243)	*A. mellifera* Buckfast (honey stomach)	1410	99.8
bbr24 (HM534853)	*A. mellifera scutellata* (honey stomach)	945	98.6
HBG-A5R3-2 (DQ837637)	*A. mellifera* (gut)	1371	99.7
Sal8 (HQ842700)	*A. mellifera mellifera* (digestive tract)	1432	99.7
***L. kimbladii* Hma2N^T^ (JX099549)**			
H6hs28N (JX099548)	*A. mellifera mellifera* (honey stomach)	1438	99.8
Hma2 (EF187240)	*A. mellifera* Buckfast (honey stomach)	1409	99.8
SHOG578 (HM113216)	*A. mellifera* (gut)	1397	99.7
AMW-G6 (HM046580)	*A cerana indica* (midgut)	1440	99.6
C1 (KF543104)	*A. cerana* (gut)	1488	99.7
hmr1 (HM534847)	*A. mellifera monticola* (honey stomach)	945	98.9
Mbopo2r2 (HM534803)	*M. bocandei* (bee pollen)	1438	99.7
Mbobb2r6 (HM534802)	*M. bocandei* (bee bread)	1438	99.7
***L. kullabergensis* Biut2N^T^ (JX099550)**			
H6hs21N (JX099547)	*A. mellifera mellifera* (honey stomach)	1438	99.7
Biut2 (EF187241)	*A. mellifera* Buckfast (honeybee)	1410	100
1G2 (AY667698)	*A. mellifera* (gut)	1451	100
SHOA503 (HM111947)	*A. mellifera* (gut)	1398	99.7
por13 (HM534849)	*A. mellifera monticola* (bee pollen)	945	99.9
bbr19 (HM534852)	*A. mellifera scutellata* (bee bread)	945	99.9
Mbohs2t2 (HM534799)	*M. bocandei* (honey stomach)	1438	99.9
Mbohs2r12 (HM534800)	*M. bocandei* (honey stomach)	1438	99.9
Mbopo2t6 (HM534801)	*M. bocandei* (bee pollen)	1438	99.8
AMW-G6 (HM046580)	*A. cerana indica* (midgut)	1526	99.5
C1 (KF543104)	*A. cerana* (gut)	1488	99.7

The 16S rRNA gene sequence of the reference strain H4bb18N showed 100.0 % similarity to that of Bma5N^T^. In the MSP dendrogram, Bma5N^T^ grouped close together with the reference strain H4bb18N, and both were well separated from the closest type strain, *L. apis* R4B^T^, and the other proposed type strains (Fig. 2). According to this analysis, strains Bma5N^T^ and H4bb18N represent a sixth novel species of the genus *Lactobacillus*, for which we propose the name *Lactobacillus helsingborgensis* sp. nov. 

Finally, the 16S rRNA gene sequence of the reference strain H6hs21N showed 99.5 % similarity to that of Biut2N^T^. In both the phylogenetic tree (Fig. 1) and the MSP dendrogram (Fig. 2), Biut2N^T^ grouped close together with the reference strain H6hs21N, but the reference strain also grouped close to the proposed novel type strain Hma2N^T^ and the reference strain H6hs28N, as mentioned previously; however, these results varied with settings in the respective software, especially in the phylogenetic tree. As mentioned above, results from API 50 tests (Table 1) clearly showed the relatedness of the reference strains to their respective proposed type strains. Both strains were well separated from the closest type strain, *L. apis* R4B^T^, and the other proposed type strains. According to our analysis, strains Biut2N^T^ and H6hs21N represent a seventh novel species of the genus *Lactobacillus*, for which we propose the name *Lactobacillus kullabergensis* sp. nov.

Sequences closely related to those of the novel strains that are available in GenBank are detailed in Table 3.

## Description of *Lactobacillus apinorum* sp. nov.

*Lactobacillus apinorum* (a.pi.no′rum. N.L. masc. pl. n. *Apini* scientific zoological name of a tribe including only the genus *Apis* and referring to honeybees; N.L. gen. masc. pl. n. *apinorum* of the Apini, referring to the isolation of strains of this species from honeybees in northern Sweden but also originating from other *Apis* species).

Cells are Gram-stain-positive, non-motile, non-spore-forming, catalase-negative rods, 0.5–0.8×1.5–6.0 µm, and occur singly or in pairs. After anaerobic growth on supplemented MRS agar (0.1 % l-cysteine and 2.0 % fructose) at 35 °C for 72 h, colonies appear white and opaque. They have a smooth to rough surface, and are circular, raised and approximately 3–4 mm in diameter. Facultatively anaerobic and grows well on supplemented MRS agar under aerobic conditions. On supplemented MRS agar, growth occurs at 15–50 °C; in supplemented MRS broth, growth occurs at pH 3.0–12.0. d-Lactate is produced as the end product from hexoses. Gas is produced from glucose. Results from API 50 and API ZYM tests (Table 1) show production of acid from d-glucose and d-fructose and enzyme activities for leucine arylamidase, valine arylamidase, acid phosphatase and naphthol-AS-BI-phosphohydrolase and weak activity for cystine arylamidase. The major fatty acids detected are C_16 : 0_, C_19 : 0_ cyclo ω8*c*, summed unknown 18.846/C_19 : 1_ω6*c*/C_19 : 0_ cyclo ω10*c/*C_19_ω6 and C_18 : 1_ω9*c*. The polar lipids comprise diphosphatidylglycerol, phosphatidylglycerol and phosphatidylethanolamine, together with an uncharacterized phospholipid, two uncharacterized phosphoaminolipids and two glycolipids. Cells do not contain m-DAP acid in their cell-wall peptidoglycan. The cell-wall peptidoglycan is of the A4α l-Lys–d-Asp type.

The type strain Fhon13N^T^ ( = DSM 26257^T^ = CCUG 63287^T^) and the reference strain Kvahm3N ( = DSM 26315 = CCUG 63288) were both isolated from the honey stomach of the honeybee *A. mellifera mellifera*. The DNA G+C content of the type strain is 34.7 mol%.

## Description of *Lactobacillus mellifer* sp. nov.

*Lactobacillus mellifer* (mel′li.fer. L. adj. *mellifer* -*fera* -*ferum* honey-bearing, honey-producing; L. masc. adj. *mellifer* intended to mean isolated from the honeybee *A. mellifera*).

Cells are Gram-stain-positive, non-motile, non-spore-forming, catalase-negative rods, 0.5–0.8×5.0–9.0 µm, and occur singly or in pairs. After anaerobic growth on supplemented MRS agar (0.1 % l-cysteine and 2.0 % fructose) at 35 °C for 72 h, colonies appear colourless, punctiform and circular with a diameter of approximately 2–3 mm. Facultatively anaerobic and grows well on supplemented MRS agar under aerobic conditions. On supplemented MRS agar, growth occurs at 15–50 °C. Growth occurs in supplemented MRS broth at pH 3.0–12.0. d-Lactate is produced as the end product from hexoses. Gas is not produced from glucose. Results from API 50 and API ZYM tests (Table 1) show production of acid from d-glucose and d-fructose, arbutin and d-arabitol and weak acid production from salicin. Aesculin is hydrolysed. Enzyme activities are shown for leucine arylamidase, valine arylamidase, acid phosphatase, naphthol-AS-BI-phosphohydrolase, cystine arylamidase, β-glucosidase, esterase and esterase lipase. The major fatty acids detected are C_18 : 1_ω9*c*, C_16 : 0_, summed unknown 18.846/C_19 : 1_ω6*c*/C_19 : 0_ cyclo ω10*c/*C_19_ω6 and summed C_18 : 1_ω7*c*/C_18 : 1_ω6*c*. The polar lipids comprise diphosphatidylglycerol and phosphatidylglycerol together with an uncharacterized phospholipid and seven glycolipids. Cells do not contain m-DAP in their cell-wall peptidoglycan. The cell-wall peptidoglycan is of the A4α l-Lys–d-Asp type. 

The type strain, Bin4N^T^ ( = DSM 26254^T^ = CCUG 63291^T^), and the reference strain Bin4 ( = DSM 26316 = CCUG 65819) were isolated from the honey stomach, but from the two different honeybees, a specimen of *A. mellifera mellifera* (Bin4N^T^) and *A. mellifera* bred according to Buckfast (Bin4). The DNA G+C content of the type strain is 39.4 mol%.

## Description of *Lactobacillus mellis* sp. nov.

*Lactobacillus mellis* (mel′lis. L. gen. n. *mellis* of honey).

Cells are Gram-stain-positive, non-motile, non-spore-forming, catalase-negative rods, 1.0–1.2×3.0–6.0 µm, and occur singly or in pairs. After anaerobic growth on supplemented MRS agar (0.1 % l-cysteine and 2.0 % fructose) at 35 °C for 72 h, colonies appear white, translucent, with a smooth to rough surface, circular, with a convex elevation, moist and punctiform, with a diameter of approximately 2–3 mm. Facultatively anaerobic and grows well on supplemented MRS agar under aerobic conditions. On supplemented MRS agar, growth occurs at 15–50 °C. Growth occurs in supplemented MRS broth at pH 3.0–12.0. d-Lactate is produced as the end product from hexoses. Gas is not produced from glucose. Results from API 50 and API ZYM tests (Table 1) show production of acid from d-glucose, d-fructose, arbutin and salicin. Aesculin is hydrolysed. Enzyme activities are shown for leucine arylamidase, valine arylamidase, acid phosphatase, naphthol-AS-BI-phosphohydrolase, cystine arylamidase, β-glucosidase, esterase and esterase lipase. The major fatty acids detected are C_18 : 1_ω9*c*, summed unknown 18.846/C_19 : 1_ω6*c*/C_19 : 0_ cyclo ω10*c/*C_19_ω6, C_16 : 0_ and summed C_18 : 1_ω7*c*/C_18 : 1_ω6*c*. The polar lipids comprise diphosphatidylglycerol, phosphatidylglycerol and phosphatidylethanolamine, together with an uncharacterized phospholipid, one uncharacterized phosphoaminolipid and five glycolipids. Cells do not contain m-DAP in their cell wall peptidoglycan. The cell-wall peptidoglycan is of the A4α l-Lys–d-Asp type.

The type strain Hon2N^T^ ( = DSM 26255^T^ = CCUG 63289^T^) and the reference strain H1hs38N ( = DSM 26313 = CCUG 63290) were isolated from the honey stomach of the honeybee *A. mellifera mellifera*. The DNA G+C content of the type strain is 36.4 mol%.

## Description of *Lactobacillus melliventris* sp. nov.

*Lactobacillus melliventris* (mel.li.ven′tris. L. n. *mel*, *mellis* honey; L. n. *venter*, -*tris* belly, stomach; N.L. gen. n. *melliventris* of the honey stomach, referring to the isolation of the first strains from the honey stomach of honeybees).

Cells are Gram-stain-positive, non-motile, non-spore-forming, catalase-negative rods, 0.5–0.8×2.0–7.0 µm, and occur singly or in pairs. After anaerobic growth on supplemented MRS agar (0.1 % l-cysteine and 2.0 % fructose) at 35 °C for 72 h, colonies appear white and opaque, with a smooth to rough surface, circular, raised, moist and punctiform, with a diameter of approximately 2–3 mm. Facultatively anaerobic and grows well on supplemented MRS agar under aerobic conditions. On supplemented MRS agar, growth occurs at 15–50 °C. In supplemented MRS broth, growth occurs at pH 3.0–12.0. d-Lactate is produced as the end product from hexoses. Gas is not produced from glucose. Results from API 50 and API ZYM tests (Table 1) show production of acid from d-glucose, d-fructose, d-mannose and gentiobiose and weak acid production from d-tagatose. Aesculin is hydrolysed. Enzyme activities are shown for leucine arylamidase, valine arylamidase, acid phosphatase, naphthol-AS-BI-phosphohydrolase, cystine arylamidase, β-glucosidase, α-glucosidase and esterase and weakly for *N*-acetyl-β-glucosaminidase. The major fatty acids detected are C_18 : 1_ω9*c*, C_16 : 0_, summed unknown 18.846/C_19 : 1_ω6*c*/C_19 : 0_ cyclo ω10*c/*C_19_ω6 and summed C_18 : 1_ω7*c*/C_18 : 1_ω6*c*. The polar lipids comprise diphosphatidylglycerol, phosphatidylglycerol and phosphatidylethanolamine, together with an uncharacterized phospholipid, one uncharacterized phosphoaminolipid and ten glycolipids. Cells do not contain m-DAP in their cell-wall peptidoglycan. The cell-wall peptidoglycan is of the A4α l-Lys–d-Asp type.

The type strain Hma8N^T^ ( = DSM 26256^T^ = CCUG 63629^T^) and the reference strain Hma8 ( = DSM 26312 = CCUG 65820) were isolated from the honey stomach of two different honeybees, specimens of *A. mellifera mellifera* (Hma8N^T^) and *A. mellifera* bred according to Buckfast (Hma8). The DNA G+C content of the type strain is 35.8 mol%.

## Description of *Lactobacillus kimbladii* sp. nov.

*Lactobacillus kimbladii* (kim.bla′di.i. N.L. gen. masc. n. *kimbladii* named after beekeeper Tage Kimblad, for his significant contributions to the discovery of the LAB microbiota in the honey stomach of honeybees).

Cells are Gram-stain-positive, non-motile, non-spore-forming, catalase-negative rods, 0.5×3.0–7.0 µm, and occur singly or in pairs. After anaerobic growth on supplemented MRS agar (0.1 % l-cysteine and 2.0 % fructose) at 35 °C for 72 h, colonies appear white and opaque, with a smooth to rough surface, circular, raised, moist and punctiform, with a diameter of approximately 2–3 mm. Facultatively anaerobic and grows well on supplemented MRS agar under aerobic conditions. On supplemented MRS agar, growth occurs at 15–50 °C. Growth occurs in supplemented MRS broth at pH 3.0–12.0. d-Lactate is produced as the end product from hexoses. Gas is not produced from glucose. Results from API 50 and API ZYM tests (Table 1) show production of acid from d-glucose, d-fructose, d-mannose, *N*-acetylglucosamine, arbutin, salicin and d-tagatose and weak acid production from maltose. Aesculin is hydrolysed. Enzyme activities are shown for leucine arylamidase, valine arylamidase, acid phosphatase and naphthol-AS-BI-phosphohydrolase and weak activity is shown for cystine arylamidase. The major fatty acids detected are C_18 : 1_ω9*c*, C_16 : 0_, summed unknown 18.846/C_19 : 1_ω6*c*/C_19 : 0_ cyclo ω10*c/*C_19_ω6 and summed C_18 : 1_ω7*c*/C_18 : 1_ω6*c*. The polar lipids comprise diphosphatidylglycerol and phosphatidylglycerol together with three uncharacterized phospholipids, four uncharacterized lipids and ten glycolipids. Cells do not contain m-DAP in their cell-wall peptidoglycan. The cell-wall peptidoglycan is of the A4α l-Lys–d-Asp type.

The type strain, Hma2N^T^ ( = DSM 26263^T^ = CCUG 63633^T^), and the reference strain H6hs28N ( = DSM 26318 = CCUG 63634) were both isolated from the honey stomach of the honeybee *A. mellifera mellifera*. The DNA G+C content of the type strain is 35.9 mol%.

## Description of *Lactobacillus helsingborgensis* sp. nov.

*Lactobacillus helsingborgensis* (hel.sing.bor.gen′sis. N.L. masc. adj. *helsingborgensis* pertaining to Helsingborg, the site of Lund University, Campus Helsingborg, Sweden, where the type strain was characterized).

Cells are Gram-stain-positive, non-motile, non-spore-forming, catalase-negative rods, 0.5–0.8×2.0–7.0 µm, and occur singly or in pairs. After anaerobic growth on supplemented MRS agar (0.1 % l-cysteine and 2.0 % fructose) at 35 °C for 72 h, colonies appeared white and opaque, with a smooth to rough surface, circular, raised, moist and punctiform, with a diameter of approximately 2–3 mm. Facultatively anaerobic and grows well on supplemented MRS agar under aerobic conditions. On supplemented MRS agar, growth occurs at 15–50 °C. In supplemented MRS broth, growth occurs at pH 3.0–12.0. d-Lactate is produced as the end product from hexoses. Gas is not produced from glucose. Results from API 50 and API ZYM tests (Table 1) show production of acid from d-glucose, d-mannose, l-sorbose, d-sorbitol, *N*-acetylglucosamine, salicin and sucrose and weak acid production from d-galactose, d-fructose, dulcitol, methyl α-d-glucoside, amygdalin, arbutin and raffinose. Aesculin is hydrolysed. Enzyme activities are shown for leucine arylamidase, valine arylamidase, acid phosphatase, naphthol-AS-BI-phosphohydrolase, β-glucosidase, β-galactosidase and esterase. The major fatty acids detected are C_18 : 1_ω9*c*, C_16 : 0_, summed unknown 18.846/C_19 : 1_ω6*c*/C_19 : 0_ cyclo ω10*c/*C_19_ω6 and summed C_18 : 1_ω7*c*/C_18 : 1_ω6*c*. The polar lipids comprise diphosphatidylglycerol, phosphatidylglycerol and phosphatidylethanolamine, together with an uncharacterized phosphoaminolipid, two uncharacterized phospholipids, two uncharacterized lipids and ten glycolipids. Cells do not contain m-DAP in their cell-wall peptidoglycan. The cell-wall peptidoglycan is of the A4α l-Lys–d-Asp type.

The type strain Bma5N^T^ ( = DSM 26265^T^ = CCUG 63301^T^) and the reference strain H4bb18N ( = DSM 26317 = CCUG 63635) were both isolated from the honey stomach of the honeybee *A. mellifera mellifera*. The DNA G+C content of the type strain is 36.3 mol%.

## Description of *Lactobacillus kullabergensis* sp. nov.

*Lactobacillus kullabergensis* (kul.la.ber.gen′sis. N.L. masc. adj. *kullabergensis* of or belonging to the nature reserve Kullaberg, where the discovery of these strains was made in 2005).

Cells are Gram-stain-positive, non-motile, non-spore-forming, catalase-negative rods, 0.5×3.0–8.0 µm, and occur singly or in pairs. After anaerobic growth on supplemented MRS agar (0.1 % l-cysteine and 2.0 % fructose) at 35 °C for 72 h, colonies appear white and opaque, with a smooth to rough surface, circular, irregular and punctiform and are approximately 2–3 mm in diameter. Facultatively anaerobic and grows well on supplemented MRS agar under aerobic conditions. On supplemented MRS agar, growth occurs at 15–50 °C. In supplemented MRS broth, growth occurs at pH 3.0–12.0. d-Lactate is produced as the end product from hexoses. Gas is not produced from glucose. Results from API 50 and API ZYM tests (Table 1) show production of acid from d-glucose, d-fructose, trehalose and gentiobiose and weak acid production from salicin. Aesculin is not hydrolysed. Enzyme activities are detected for leucine arylamidase, valine arylamidase, acid phosphatase, naphthol-AS-BI-phosphohydrolase and β-glucosidase and weak activity of esterase and *N*-acetyl-β-glucosaminidase is detected. The major fatty acids detected are C_18 : 1_ω9*c*, C_16 : 0_, summed unknown 18.846/C_19 : 1_ω6*c*/C_19 : 0_ cyclo ω10*c/*C_19_ω6 and summed C_18 : 1_ω7*c*/C_18 : 1_ω6*c*. The polar lipids comprise diphosphatidylglycerol, phosphatidylglycerol and phosphatidylethanolamine, together with an uncharacterized phosphoaminolipid, two uncharacterized phospholipids and ten glycolipids. Cells do not contain m-DAP in their cell-wall peptidoglycan. The cell-wall peptidoglycan is of the A4α l-Lys–d-Asp type.

The type strain Biut2N^T^ ( = DSM 26262^T^ = CCUG 63631^T^) and the reference strain H6hs21N ( = DSM 26314 = CCUG 63632) were isolated from the honey stomach of the honeybee *A. mellifera mellifera*. The DNA G+C content of the type strain is 35.6 mol%.
